# Enhanced Image Annotation in Wild Blueberry (*Vaccinium angustifolium* Ait.) Fields Using Sequential Zero-Shot Detection and Segmentation Models

**DOI:** 10.3390/s25237325

**Published:** 2025-12-02

**Authors:** Connor C. Mullins, Travis J. Esau, Riley Johnstone, Chloe L. Toombs, Patrick J. Hennessy

**Affiliations:** 1 Department of Engineering, Faculty of Agriculture, Dalhousie University, Truro, NS B2N 5E3, Canada; cmullins@dal.ca (C.C.M.); patrick.hennessy@dal.ca (P.J.H.); 2 Atlantic Institute for Digital Agriculture, Dalhousie University, Truro, NS B2N 5E3, Canada

**Keywords:** Grounding DINO, YOLO-world, grounded SAM, SAM2, deep learning

## Abstract

This research addresses the critical need for efficient image annotation in precision agriculture, using the wild blueberry (*Vaccinium angustifolium* Ait.) cropping system as a representative application to enable data-driven crop management. Tasks such as automated berry ripeness detection, plant disease identification, plant growth stage monitoring, and weed detection rely on extensive annotated datasets. However, manual annotation is labor-intensive, time-consuming, and impractical for large-scale agricultural systems. To address this challenge, this study evaluates an automated annotation pipeline that integrates zero-shot detection models from two frameworks (Grounding DINO and YOLO-World) with the Segment Anything Model version 2 (SAM2). The models were tested on detecting and segmenting ripe wild blueberries, developmental wild blueberry buds, hair fescue (*Festuca filiformis* Pourr.), and red leaf disease (*Exobasidium vaccinii*). Grounding DINO consistently outperformed YOLO-World, with its Swin-T achieving mean Intersection over Union (mIoU) scores of 0.694 ± 0.175 for fescue grass and 0.905 ± 0.114 for red leaf disease when paired with SAM2-Large. For ripe wild blueberry detection, Swin-B with SAM2-Small achieved the highest performance (mIoU of 0.738 ± 0.189). Whereas for wild blueberry buds, Swin-B with SAM2-Large yielded the highest performance (0.751 ± 0.154). Processing times were also evaluated, with SAM2-Tiny, Small, and Base demonstrating the shortest durations when paired with Swin-T (0.30–0.33 s) and Swin-B (0.35–0.38 s). SAM2-Large, despite higher segmentation accuracy, had significantly longer processing times (significance level α = 0.05), making it less practical for real-time applications. This research offers a scalable solution for rapid, accurate annotation of agricultural images, improving targeted crop management. Future research should optimize these models for different cropping systems, such as orchard-based agriculture, row crops, and greenhouse farming, and expand their application to diverse crops to validate their generalizability.

## 1. Introduction

Image annotation is pivotal in various agricultural practices, including creating prescription maps, identifying plant species and diseases, and analyzing growth stages [1,2,3,4]. With advancements in machine learning and computer vision, the demand for rapid and accurate data annotation has increased. Training machine learning models often requires thousands of precisely annotated images, a task that can be time-consuming [5,6]. In wild blueberry (*Vaccinium angustifolium* Ait.) cultivation, image annotation aids in detecting ripe berries, monitoring growth stages, and identifying disease infections and weeds. Accurate fruit identification supports yield estimation, quality assessment, and ripeness determination [7,8]. However, this task requires experienced personnel trained in feature recognition. The ability to identify weeds and diseases allows management practices such as variable rate application of agrochemicals to be used [9,10]. Applied at various stages during wild blueberry growth, agrochemicals such as herbicides and fungicides are essential for weed and disease prevention [11,12]. Identifying the bud stage, crucial for optimal fungicide application, can be challenging for inexperienced personnel but is essential for maximizing yield.

Wild blueberries are grown in a 2-year cropping cycle with the first year being the sprout year and the second year being the harvest year [13]. Throughout these two years, fungicides, herbicides and insecticides are applied at specific times within the cropping cycle based on the growth stage of the wild blueberry plants [14,15]. Growth stages along with other factors such as growing degree days are also used for determining the correct timings for pollination and harvest [15]. The percentage of buds in F2 stage of development can help producers ensure proper timing when managing their fields [16]. Approximately 5% of a wild blueberry field is in the F2 stage at 110 growing degree days while 80% of the field is in the F2 stage at 222 growing degree days [16]. Wild blueberry fields are harvested at approximately 1700 growing degree days with the first ripening occurring around 1142 growing degree days [16].

Wild blueberries are susceptible to numerous diseases that significantly impact yield and quality [17,18]. These diseases can originate from environmental conditions or spread from nearby plants [17,18]. Identifying these diseases is crucial for preventing them in future cropping cycles. While some diseases are easily identifiable, others require expert identification [19,20]. Red Leaf disease (*Exobasidium vaccinii*), a common foliar disease, is characterized by red or brown leaf discoloration and significantly reduces yield [17,21]. Accurate weed identification also optimizes treatment application, reducing environmental impact and costs. Hair fescue (*Festuca filiformis* Pourr.), prevalent in 75% of wild blueberry fields in Nova Scotia, significantly reduces crop yield [12,22,23]. Traditional broadcast spraying of agrochemicals can be replaced with spot spraying by precisely identifying weed locations, offering environmental and economic benefits [24]. These challenges highlight the need for precise identification and management of diseases and weeds in wild blueberry fields, underscoring the importance of advanced image annotation techniques. However, existing methods for image annotation in precision agriculture are limited by their reliance on manual or semi-automated approaches, which are labor-intensive and often lack consistency in complex field environments.

Zero-shot detection methods have become pivotal in addressing the challenges in agricultural image annotation [25,26]. These methods enable the identification and segmentation of objects without pre-labeled data, which is advantageous in agriculture where manual annotation can be time consuming and costly. Mullins et al. (2024) [26] evaluated Grounding DINO and YOLO-World, on datasets relevant to wild blueberry cultivation. Grounding DINO with its strong visual representation learning and language-grounded pre-training demonstrated a superior ability to generalize to new object categories specified by textual descriptions, outperforming YOLO-World in several key metrics. YOLO-World combines the efficiency of YOLOv8′s real-time detection capabilities with advanced vision-language pre-training [27]. Across all datasets, Grounding DINO outperformed YOLO-World on metrics including Intersection over Union (IoU), precision, recall, and F1 score, making it a valuable tool for precision agriculture applications that require precise object detection [26]. By leveraging these advanced models, producers can achieve more accurate and efficient identification of diseases, weeds, and growth stages in wild blueberry fields, thereby improving crop management [26].

The task of segmentation within machine learning offers substantial potential for reducing annotation requirements through automated labeling. Unlike object detection, which uses bounding boxes, segmentation annotation requires drawing polygon segmentation masks [28,29]. The Segment Anything Model (SAM) represents a substantial advancement in computer vision, particularly in image segmentation [30,31]. SAMs were developed utilizing a combination of supervised and self-supervised learning mechanisms, achieving remarkable precision in segmenting both common and uncommon objects [30,32]. SAM’s architecture is built around a Vision Transformer (ViT) image encoder, a prompt encoder, and a lightweight mask decoder [30]. The ViT encoder generates image embeddings, while the prompt encoder processes user-provided inputs such as points, boxes, or coarse masks. These embeddings are then fused by the mask decoder to produce high-resolution segmentation outputs. This modular design allows for flexibility and adaptability across segmentation tasks, enabling the tailoring of components to specific application requirements, including agricultural contexts.

Grounded SAM is a powerful tool that integrates the capabilities of Grounding DINO and SAM to excel in both object detection and segmentation tasks [33]. The model begins with feature extraction using the network’s backbone, employing attention mechanisms to focus on relevant parts of the image, enhancing the accuracy of detection and segmentation. Grounded SAM processes images at multiple scales to capture objects of different sizes, ensuring that both small and large objects are accurately detected and segmented. Grounded SAM leverages the strengths of Grounding DINO, predicting bounding boxes and class labels for each detected object. Once objects are detected, the model applies the semantic segmentation techniques from SAM to generate detailed segmentation maps [33]. The integration of contextual information from the entire image further improves segmentation quality, allowing the model to accurately segment objects in complex scenes.

Existing methods for image annotation in precision agriculture face several limitations. Traditional manual and semi-automated approaches are labor-intensive, time-consuming, and often lack consistency, particularly in complex field environments [34,35]. While zero-shot learning models have recently demonstrated capabilities in general-purpose detection and segmentation tasks, their application to agriculture remains largely unexplored [36], with wild blueberry applications in particular being completely unexplored. There is a critical need for innovative adaptations of these advanced models to the unique challenges of agricultural environments, particularly for improving the efficiency and accuracy of instance segmentation. Zero-shot and openset detection and segmentation have seen growing applications in computer vision, including in agricultural contexts such as plant disease identification, pest detection, and crop phenotyping [37,38,39]. However, existing agricultural studies have treated zero-shot detection and segmentation as separate stages or rely on fixed prompt sets, rather than integrating dynamic prompt tuning and segmentation refinement in a unified pipeline. To date, the use of Grounding DINO and YOLO-World sequentially with SAM2 has not been evaluated in agriculture. Furthermore, no studies have been conducted using this approach to identify multiple targets in the wild blueberry cropping system.

The objective of this research was to address the challenge of efficient image annotation in precision agriculture by developing and validating an automated annotation pipeline leveraging zero-shot learning. This study integrates Grounding DINO and YOLO-World sequentially with the SAM2 segmentation framework to enhance instance segmentation annotation efficiency. The approach is tailored to identifying diverse agricultural targets within wild blueberry fields, including ripe wild blueberries (“Berry”), F2 developmental buds (“Bud”), hair fescue grass (“Fescue”), and red leaf disease infected plants (“Red leaf”). By systematically optimizing and comparing these models, this research establishes a novel, replicable pipeline for automated instance segmentation in complex field environments.

While previous work [26] evaluated zero-shot object detection frameworks on wild blueberry datasets, it focused exclusively on generating bounding box annotations. In contrast, the present study introduces a novel sequential pipeline that combines zero-shot detection (Grounding DINO and YOLO-World) with SAM2 for detailed instance segmentation. Additionally, this work includes an extensive optimization of textual prompts and confidence thresholds to maximize detection and segmentation accuracy. By integrating segmentation and refining prompt strategies, this approach advances beyond basic object detection to enable fully automated, high-resolution instance annotations suitable for complex agricultural environments.

The innovation of this work lies in several key aspects. It represents the first application of sequential zero-shot detection and segmentation models to multiple agricultural targets, addressing unique challenges such as variable lighting, foliage, hardware, and target variability. The research also introduces an optimization pipeline for prompt tuning and confidence adjustments, improving performance. Through an evaluation of interactions between Grounding DINO and YOLO World with four SAM2 models (Tiny, Small, Base, and Large), the study identifies optimal combinations that balance accuracy and efficiency. Specific objectives of this research include: (1) To apply zero-shot learning models to detect and segment multiple agricultural targets in wild blueberry fields. (2) To optimize and compare model performance, determining the most effective configurations for accurate and efficient annotation.

## 2. Materials and Methods

### 2.1. Dataset Preparation

**Figure 1:**
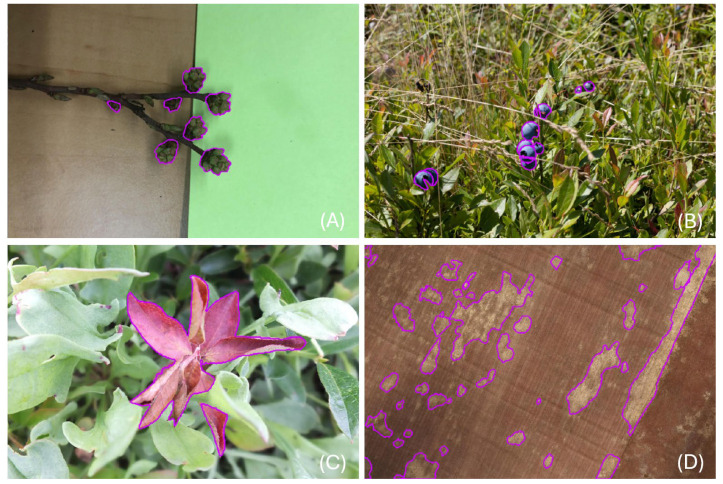
Examples from each dataset with ground truth segmentation masks overlayed (magenta) where (**A**) shows F2 stage developmental buds, (**B**) shows ripe wild blueberries, (**C**) shows the red leaf disease that can be found in wild blueberry fields, and (**D**) shows hair fescue grass in a sprout stage wild blueberry field.

The dataset used in this study comprised four target classes: developmental buds (F2 stage) (Figure 1A), ripe wild blueberries (Figure 1B), red leaf disease (Figure 1C), and hair fescue (Figure 1D) [26]. Image acquisition included controlled laboratory setups, ground-based field photography, and aerial drone surveys, summarized in Table 1, spanning from June of 2022 to May of 2024. The drone flight altitude of 76 m was selected to ensure sufficient ground resolution while maximizing field coverage, consistent with established imaging protocols in precision agriculture. Surveying at an altitude of 76 m resulted in a spatial resolution of 8.63 mm. Ground truth annotations, including both bounding boxes and polygon masks, were prepared using Roboflow’s polygon tool (Roboflow, Inc., Des Moines, IA, USA) and used for evaluating model performance. Representative examples for each class are shown in Figure 1.

### 2.2. Model Selection

For the initial detection stage within the pipeline, state-of-the-art zero-shot learning models, specifically ViTs and contrastive language-image pre-training (CLIP)-based models were selected for their robust performance in zero-shot scenarios [40]. Grounding DINO, utilizing ViTs, and YOLO-World, using CLIP for encoder pretraining, were chosen for comparison. Grounding DINO’s Swin-T and Swin-B variants were studied to assess the trade-offs between computational efficiency and detection performance, with Swin-T designed for real-time processing and Swin-B offering higher capacity for better feature extraction. The YOLO-World framework included four variants (YOLO-World-s, YOLO-World-m, YOLO-World-l, YOLO-World-x) to evaluate the impact of model complexity and size on annotation performance.

Four variants of the SAM2 were used in conjunction with Grounding DINO and YOLO-World frameworks to assess their combined performance: SAM2-Tiny, SAM2-Small, SAM2-Base, and SAM2-Large. The SAM2 variants differ in model size (measured in millions of parameters), segmentation accuracy, and computational requirements. Tiny and Small are designed for faster processing and lower resource use, making them suitable for real-time or edge applications. Base provides a balance between accuracy and efficiency, while Large offers the highest segmentation precision but at increased computational cost. These characteristics are summarized in Table 2.

**Figure 2:**
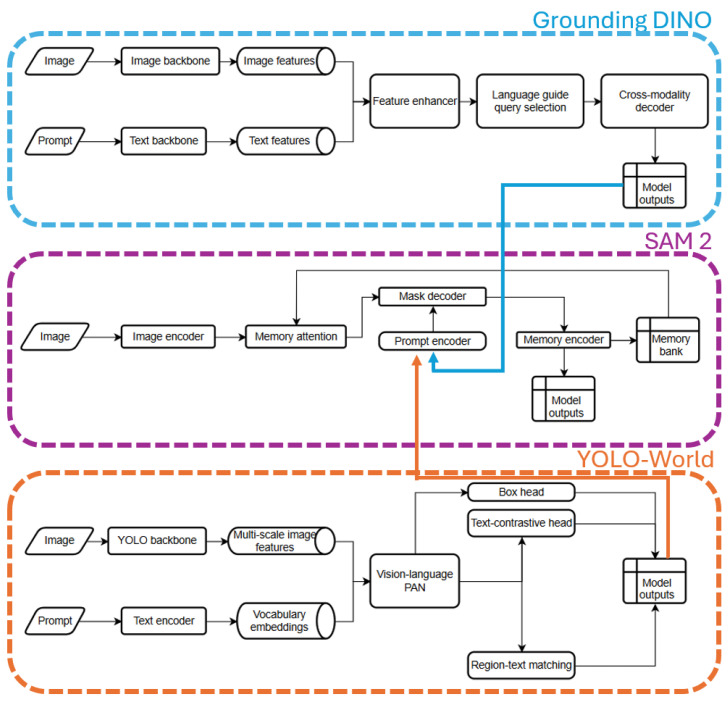
System architecture diagram of the three core modules used in the annotation pipeline: Grounding DINO (**top**), SAM2 (**middle**), and YOLO-World (**bottom**). Each diagram illustrates the model-specific flow of image and prompt inputs through their respective backbones, encoders, and attention mechanisms, leading to detection or segmentation outputs.

To provide further clarity on the underlying system structure, an architecture diagram (Figure 2) outlines the internal design of the selected models used in the annotation pipeline. The architecture diagram visualizes the individual data pathways for both image and text prompts, the feature extraction and enhancement stages, and the processes through which model outputs are generated.

### 2.3. Optimization and Tuning

To increase the performance of the zero-shot models several steps were taken to optimize the input prompts and confidences. A list of 100 potential prompts for each category was created, through a combination of human input and the GPT-4o Large Language Model (LLM) (OpenAI, San Francisco, CA, USA, 2024), followed by removal of any duplicate or inaccurate prompts of the given category. For each category, every prompt in the list was tested with a preset confidence of 0.30 for Grounding DINO Swin-T and 0.001 for YOLO-World-l. Using the ten highest mean Intersection over Union (mIoU) [30] yielding prompts, the prompts and confidence were further optimized. For each of the ten prompts, the confidence was optimized to two significant figures, if the optimal confidence was below 0.01 the process would continue to lower, to a maximum of five decimal places of resolution. The prompt and confidence pair with the highest mIoU was selected for testing in the respective category. This process was completed separately for each object detection model in each category.

For hair fescue, red leaf and buds inferenced using YOLO-World, many images exhibited classification, or bounding boxes comprising more than 90% of the image area. To alleviate this, a label cleaning algorithm was created which removes all bounding boxes over a specified area threshold, exceptions were made with images only containing one bounding box. The box threshold was set to 0.4 for berries and red leaf, 0.5 for fescue, and 0.25 for buds. For fescue, the high-resolution drone images were tiled into 8 × 8 chunks, resulting in image dimensions is 1024 × 682, using the preprocessing option in Roboflow’s dataset generation, as the full-size images (8192 × 5460 pixels) are too difficult for the zero-shot models to discern target boundaries effectively due to the relative size and target density [26]. The text threshold, the minimum confidence for a token to be considered a detection in Grounding DINO, was set to 0.1 and remained unchanged.

**Figure 3:**
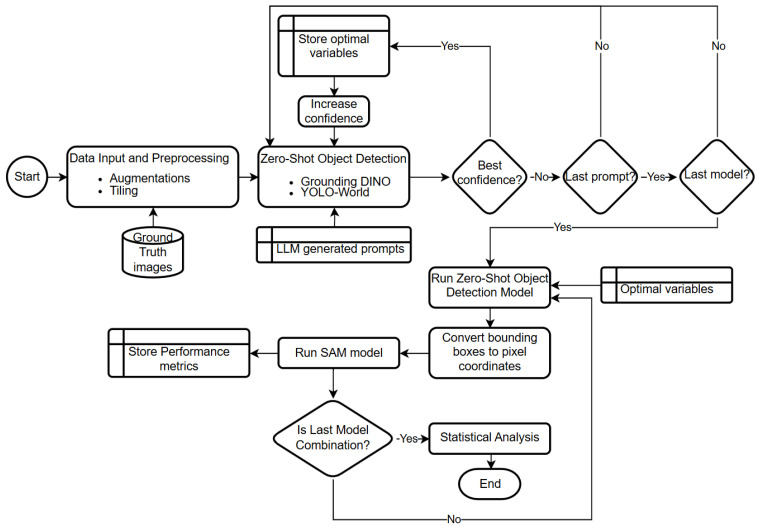
Overview of the automated annotation and optimization pipeline integrating zero-shot object detection, LLM-guided prompt generation, confidence tuning, and SAM segmentation, followed by statistical performance evaluation.

A detailed flowchart of the proposed annotation and optimization pipeline, summarizing data preprocessing, prompt generation, iterative confidence tuning, zero-shot object detection, segmentation integration, and statistical evaluation, is provided in Figure 3 to illustrate the full methodological sequence.

### 2.4. SAM Automatic Annotation Integration

Bounding boxes were passed into SAM2, so that only the area within each box was segmented. Grounding DINO outputs boxes in a format where the normalized x-position, y-position, width and height of each box is listed in the specified order. It was necessary to convert these boxes to the format accepted by SAM2 which consists of the x and y coordinates (in pixels) of the top-left corner of the box, followed by the x and y-coordinates of the bottom-right corner.

### 2.5. Evaluation Procedure

Both the zero-shot detection and segmentation models were deployed without additional training. Each model was evaluated on an Alienware Aurora R11 desktop computer (Dell Inc., Round Rock, TX, USA) with 3.7 GHz 10-core Intel Core i9-10900K CPU (Intel Corporation, Santa Clara, CA, USA), 128 GB of DDR4-3200 MT/s RAM and an Nvidia GeForce RTX 3090 GPU (Nvidia Corp., Santa Clara, CA, USA). Grounding DINO, YOLO-World, and SAM2 models were assessed on the same datasets to ensure consistent evaluation. IoU and processing time were calculated and recorded for each image within each dataset. The IoU is crucial in object detection and instance segmentation tasks, as it quantifies the overlap between the predicted and actual bounding boxes/polygons, providing a spatial accuracy assessment [42].

### 2.6. Statistical Analysis

After conducting the evaluations, the performance metrics were subjected to statistical analysis to determine any significant differences between the detection model and SAM2 combinations. A 6x4 factorial design was employed, involving two primary factors: detection model, with six levels (Grounding DINO Swin-T, Grounding DINO Swin-B, YOLO-World-s, YOLO-World-m, YOLO-World-l, YOLO-World-x), and SAM2, with four levels (Tiny, Small, Base, Large). Each combination of these factors was tested with six replicates, resulting in a total of 144 observations. The replicates were selected using simple random sampling, creating subsets of the entire dataset. The images used for each replicate were the same across model combinations. The observations were analyzed through an ANOVA general linear model to assess the main and interaction effects on segmentation performance metrics of mIoU and processing time [43]. Following the ANOVA, multiple mean comparisons were performed using Tukey’s Honest Significant Difference (HSD) test using Minitab 21 (Minitab, LLC., Minitab 21, State College, PA, USA) statistical software with an acceptance level α of 0.05 [43]. This approach allowed for the identification of significant differences between the means of the performance metrics for different model combinations. In addition to the statistical analyses, eta squared (η^2^) was recorded as a measure of effect size for the ANOVA analyses. The use of CIELAB color space’ perceptual lightness (L*) image brightness metric was calculated at the pixel level for each image in each category to find the average L* and deviation of L* across images in each category.

## 3. Results and Discussion

The preliminary optimization of the zero-shot detection models found Grounding DINO and YOLO-World models preferred different text inputs for label optimization (Table 3). This was similar in confidence threshold optimization where Grounding DINO Swin-T typically achieved a higher confidence threshold compared to Swin-B. Similarly, YOLO-World models demonstrated varying confidence thresholds (Table 3).

The variation in textual prompts and confidence thresholds was determined through empirical optimization to maximize mIoU for each category. Larger models such as Swin-T and Swin-B could leverage more complex relationships with prompts due to their advanced feature extraction and higher capacity, enabling precise differentiation of similar targets. In contrast, YOLO-World models required extremely low confidence thresholds (0.00–4.00%) to ensure sufficient object detection coverage, reflecting their design for real-time efficiency rather than nuanced language-vision alignment. These differences highlight the impact of model architecture on prompt sensitivity and confidence calibration during open-vocabulary annotation tasks.

The optimized prompts and confidences were subsequently utilized for detection boxes supplied to the SAM2 model. The results of the bud detection indicated that the Swin-B model, when paired with any SAM2 model, achieved the highest performance in terms of mIoU (Table 4). Although differences among SAM2 models were minimal, a significant difference was noted in the zero-shot detection models. In these cases, each SAM2 model, when used sequentially with the Grounding DINO detection models, exhibited superior performance (mIoU > 0.641) compared to the YOLO-World models (mIoU (excluding model YOLO-World-x) < 0.479). The analysis of variance (ANOVA) using a general linear model revealed that the SAM2 model had no significant effect (*p* = 0.986), whereas the detection model had a significant effect (*p* < 0.001). Furthermore, there was no significant interaction effect between the SAM2 models and the detection models (*p* = 1.000).

From Table 4, the recommended models for developmental bud stage segmentation were the Grounding DINO with Swin-B and any SAM2 model. The Swin-B model consistently achieved the highest mIoU across various SAM2 model configurations, making it suitable for accurate segmentation. Given the statistically insignificant performance differences between SAM2 models (*p* = 0.986), practical considerations like computational resources and processing efficiency become crucial. The SAM2-Tiny model, despite being lightweight, does not compromise performance and is recommended for reducing compute requirements and processing time.

When evaluated on ripe wild blueberry images, the SAM2-Tiny model achieved mIoU values ranging from 0.631 to 0.731 (Table 5), with the highest performance observed in the Swin-B variant at 0.731 (±0.200). The SAM2-Small model also showed strong performance, achieving the highest mIoU with the Swin-B variant at 0.738 (±0.189). SAM2-Small exhibited lower variability across detection models compared to SAM2-Large, suggesting greater consistency and reliability. This makes SAM2-Small a strong candidate for applications requiring stability. The SAM2-Base model demonstrated mIoU values from 0.631 to 0.723, with its peak performance also with the Swin-B variant at 0.723 (±0.202). Both SAM2-Tiny and SAM2-Small consistently outperformed other models, indicating superior segmentation performance for ripe blueberries. The SAM2-Large model reached its highest mIoU with the Swin-B variant at 0.738 (±0.194). Despite the differences in mIoU values (~10%), the large standard deviations resulted in the ANOVA general linear model indicating that the main effects and interaction effects were not significant (*p* > 0.594).

All SAM2 model variants (Tiny, Small, Base, and Large) demonstrated effective segmentation capabilities, as indicated by their mIoU values. The Swin-B zero-shot detection model variant significantly consistently outperformed other model sizes across all SAM2 variants, emphasizing its robust feature extraction and detection capabilities, which are crucial for optimal segmentation of ripe wild blueberries. Although the YOLO-World model variants demonstrated incremental improvements in mIoU values with increasing model sizes, these differences were not statistically significant. This indicates that scaling up YOLO-World models results in marginal performance gains. Therefore, the selection of YOLO-World model sizes should be guided by factors such as computational efficiency and resource availability rather than expectations of significant improvements in segmentation performance.

When evaluated on images depicting red leaf disease, the SAM2-Tiny model exhibited mIoU values ranging from 0.245 to 0.884, with its highest performance observed with the Swin-T model variant, achieving an mIoU of 0.884 (±0.110) (Table 6). There was considerable variation in performance across different zero-shot detection model sizes, suggesting that the choice of detection model significantly impacts the segmentation accuracy. The SAM2-Small model performed similarly, with mIoU values ranging from 0.280 to 0.885, reaching its peak with the YOLO-World-x variant at 0.885 (±0.097), indicating a strong ability to handle red leaf disease segmentation consistently across different detection models. The SAM2-Base model showed mIoU values from 0.223 to 0.843, with the best performance recorded with the Swin-B variant at an mIoU of 0.843 (±0.212). This model, like SAM2-Tiny, demonstrated significant performance variation depending on the zero-shot detection model used. The SAM2-Large model achieved mIoU values ranging from 0.351 to 0.905, with its highest mIoU observed with the Swin-T variant at 0.905 (±0.114), underscoring its enhanced capacity to manage complex segmentation tasks. Across all SAM2 variants, the Swin-T and Swin-B detection models consistently resulted in the highest mIoU scores, emphasizing their robust feature extraction capabilities.

The broader range of mIoU values for models applied to red leaf disease images compared to ripe wild blueberries suggests that segmenting diseased leaf areas may present additional challenges. Despite notable disparities in mIoU values, large standard deviations across different model combinations resulted in no significant differences identified. ANOVA results indicated that the detection model had a significant effect on mIoU performance (*p* < 0.001), implying that the choice of zero-shot detection model significantly impacts segmentation accuracy for red leaf disease images. In contrast, neither the SAM2 model variant nor the interaction effects significantly affected mIoU performance (*p* > 0.421).

This analysis was crucial for evaluating the effectiveness of various model configurations in accurately segmenting diseased leaf areas, a key aspect of precision agriculture and plant disease management. Notably, the Swin-T and Swin-B zero-shot detection models consistently achieved the highest mIoU scores across all SAM2 variants with the exception of YOLO-World-x in combination with SAM2-Small which achieved the highest. The consistently high mIoU scores demonstrated robust feature extraction and detection capabilities ideal for segmenting red leaf disease. The Swin-T model variant achieved the highest mIoU values for the Grounding DINO framework for SAM2-Tiny (0.884 ± 0.110), SAM2-Small (0.878 ± 0.112), and SAM2-Large (0.905 ± 0.114), showcasing its effectiveness across different configurations. Similarly, the Swin-B model performed exceptionally well, with high mIoU scores for SAM2-Tiny (0.866 ± 0.135), SAM2-Small (0.867 ± 0.130), and SAM2-Large (0.887 ± 0.131), and the highest SAM2-Base score (0.843 ± 0.212).

In contrast, the YOLO-World models exhibited more variable performance, with mIoU values differing significantly across SAM2 variants. YOLO-World-m, in particular, showed relatively low mIoU values across all SAM2 variants, indicating less reliability for segmenting red leaf disease compared to Swin models. Given the non-significant performance differences among SAM2 models, the choice of SAM2 variant can be optimized based on computational efficiency and resource availability. The high variability in mIoU performance among YOLO-World model variants underscores the importance of careful selection and validation of detection models in practical applications. While some configurations may achieve high accuracy, others may perform poorly, leading to inconsistent results in real-world settings.

The results of the hair fescue segmentation indicated that the Swin-T model achieved the highest mIoU (0.694), significantly outperforming the other models across all SAM2 variants, with the SAM2-Large model achieving the peak performance of 0.694 (±0.175) (Table 7). This indicates that the Swin-T model is particularly adept at differentiating hair fescue from the surrounding blueberry plants in aerial imagery. The Swin-B model also showed strong performance, achieving its highest mIoU score of 0.647 (±0.223) with the SAM2-Large model. These results indicate that both Swin-T and Swin-B models are robust and effective for high-accuracy segmentation tasks in complex agricultural environments. In contrast, the YOLO-World models performed poorly across all SAM2 variants, with significantly lower mIoU scores. The highest mIoU within the YOLO-World framework was 0.127 (±0.083) for the YOLO-World-m model paired with the SAM2-Tiny variant. This consistent underperformance suggests that the YOLO-World models are not suitable for the precise segmentation tasks required in precision agriculture, likely due to their inability to effectively capture and differentiate the intricate features present in aerial images of hair fescue in blueberry fields.

The nature of the drone images used in this study substantially impacts model performance. Aerial imagery presents several challenges for segmentation models: the images contain complex backgrounds with varying textures and colors, making it difficult for models to distinguish between fescue and surrounding vegetation or soil; variations in lighting and the presence of shadows can create additional noise, complicating the segmentation task; drone images cover large areas with high resolution, capturing both macro and micro-level details. The Swin-T model’s superior performance suggests it effectively manages these challenges, including resolution, varying light, and complex backgrounds. However, the substantial decrease in mIoU performance from bounding box-level assessment to polygon assessment indicates that the SAM2 models are a more erroneous component in the pipeline, showing more than a 10% mIoU loss. In contrast, the YOLO-World models produced inadequate segmentation results, rendering them unsuitable for practical agricultural applications.

Across all datasets, the Swin-T and Swin-B models consistently outperformed the YOLO-World models. The Swin-T model exhibited the highest mIoU scores across different datasets, demonstrating superior feature extraction and detection capabilities. This trend was most evident in the diseased plant dataset, where the Swin-T model achieved an mIoU of 0.905 when paired with the SAM2-Large variant. This indicated the model’s exceptional ability to handle complex image features associated with disease symptoms, outperforming other datasets where the Swin-T model was still robust, but had lower mIoU scores. In comparing the developmental buds and ripe blueberries datasets, the performance of Swin-T and Swin-B models remained consistently high. However, Swin-B achieved the highest mIoU scores, slightly higher in the developmental buds (0.751) compared to the ripe blueberries dataset (0.738). This marginal difference suggests that the Swin-B model is highly effective across different stages of crop maturity. The Swin-T model also performed well in these datasets, though it was consistently behind the Swin-B model. The fescue dataset provided another perspective on model performance, where the Swin-T model led in mIoU scores. However, the performance gap between Swin-T and Swin-B was larger, indicating that the fescue dataset might present more complexity in features compared to ripe blueberries or developmental buds, allowing Swin-B to perform relatively lower to Swin-T. The YOLO-World models consistently underperformed across all datasets, showing significantly lower and more variable mIoU scores. Despite slight improvements with increased model size, the YOLO-World models failed to achieve competitive performance, emphasizing the need for more robust detection models like Swin-T and Swin-B. Additional segmentation performance metrics—including F1-score, precision, and recall for every detection–segmentation model pairing—are provided in Appendix A (Table A1, Table A2, Table A3 and Table A4) to complement the mIoU results summarized in Table 4, Table 5, Table 6 and Table 7.

An overarching finding across dataset comparisons was the choice of SAM2 model having an insignificant impact on segmentation performance when paired with the high-performing Swin-T and Swin-B models, suggesting that practical considerations such as computational efficiency and resource availability should guide their selection. This consistency across datasets implies that SAM2 models are reliably interchangeable when used with effective detection models, further simplifying the decision-making process for model deployment.

Given the insignificant differences in segmentation performance of the SAM2 model component, examining processing time helps identify the optimal combination of SAM2 and zero-shot detection models (Table 8). SAM2-Tiny and Small had the shortest processing times, with Swin-T at 0.30 ± 0.10 s and Swin-B at 0.35 ± 0.11 s and 0.36 ± 0.10 s, indicating high efficiency. In contrast, SAM2-Tiny paired with YOLO-World-x required more time, averaging 0.64 ± 0.11 s. SAM2-Base exhibited similar processing times with Grounding DINO model, where Swin-T took 0.33 ± 0.10 s and Swin-B took 0.38 ± 0.10 s. The longest time within the SAM2-Base group was for YOLO-World-l, at 1.03 ± 0.57 s. SAM2-Large had the most extended processing times, ranging from 0.42 ± 0.11 s for Swin-T to 1.23 ± 0.54 s for YOLO-World-l. Notably, all YOLO-World variants within the SAM2-Large group exceeded 0.8 s per image, indicating higher computational demands. These results suggest that SAM2-Tiny or Small, especially when paired with Swin-T or Swin-B, offers the best balance of efficiency and performance. For applications where processing time is critical, Swin-T and Swin-B models with SAM2-Tiny or Small are optimal choices, providing effective segmentation with minimal computational overhead.

The results from Table 8 show a clear distinction in processing times among the SAM2 models and zero-shot detection variants. SAM2-Tiny and Small, particularly when paired with Swin-T and Swin-B, demonstrated the shortest processing times, highlighting their computational efficiency, which is beneficial for real-time applications where speed is crucial. SAM2-Large showed the longest processing times across all model variants, with particularly high values for all YOLO-World models, suggesting that SAM2-Large, despite potentially offering higher segmentation accuracy, imposes a significant computational burden that may limit its practicality in time-sensitive applications. The differences in processing times emphasize the importance of selecting model combinations based on specific operational needs. For applications requiring rapid processing, SAM2-Tiny or SAM2-Small with Swin-T or Swin-B is recommended. Conversely, for scenarios where processing time is less critical, but accuracy is paramount, SAM2-Large with higher-performing detection models may be appropriate.

### 3.1. Qualitative Visualization and Prompt-Specific Observations

**Figure 4:**
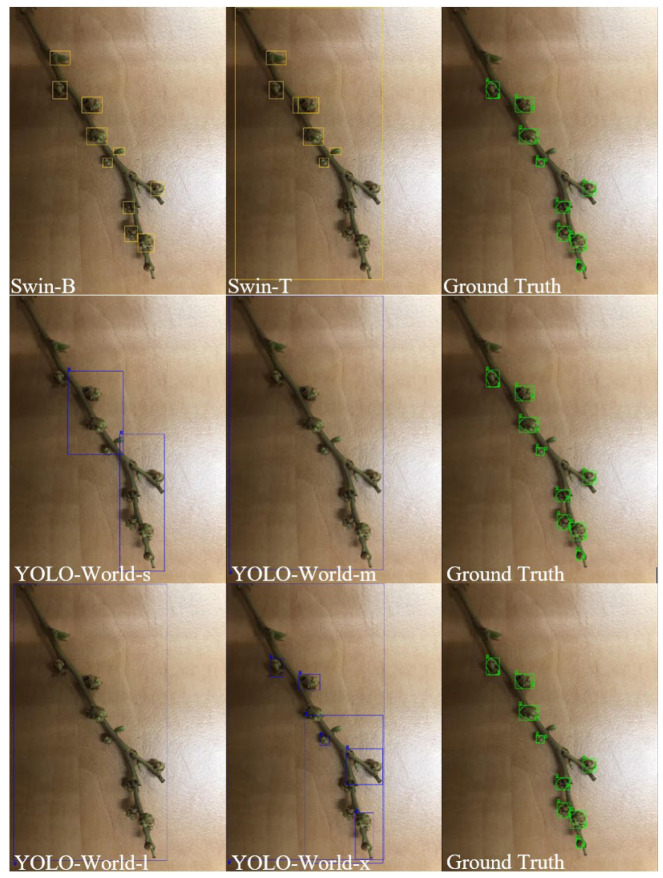
Bounding box detections for developmental buds using Swin-B and Swin-T in yellow, and YOLO-World-s, YOLO-World-m, YOLO-World-l, and YOLO-World-x configurations in blue compared to ground truth in green, illustrating differences in detection precision and segmentation outcomes.

Bounding box detections and corresponding SAM-based segmentations for developmental buds, ripe wild blueberries, and leaf regions reveal distinct differences in detection granularity and segmentation precision across model configurations. For developmental buds (Figure 4), the Swin-B configuration consistently produced a higher count of precise detections, effectively identifying individual buds along the stem while maintaining tight bounding box alignment. In contrast, Swin-T tended to under-detect smaller buds and occasionally generalized by grouping nearby buds within broader regions. The YOLO-World models demonstrated size-dependent behavior, where the smaller configuration (YOLO-World-s) occasionally fragmented clusters, while YOLO-World-m and YOLO-World-l variants included surrounding branch structures. These results indicate that transformer-based backbones (Swin) prioritize finer detail extraction, whereas YOLO-World configurations leverage broader spatial detection, influencing detection comprehensiveness. All models occasionally identified leaf buds in addition to blueberry developmental buds, suggesting shared visual cues among morphological stages.

**Figure 5:**
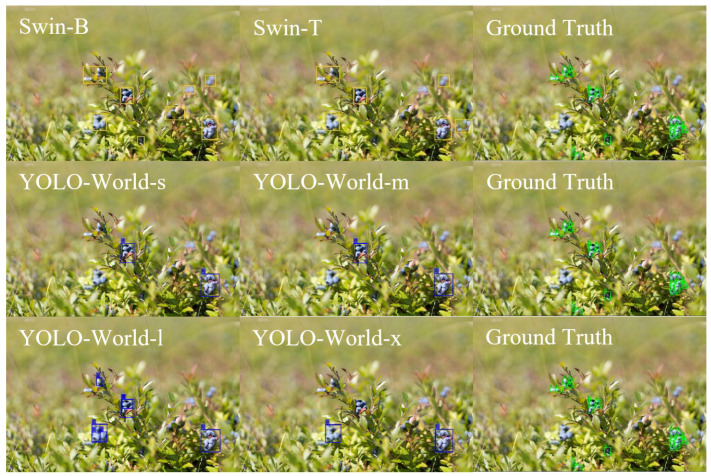
Bounding box detections for ripe wild blueberries using Swin-B, Swin-T, YOLO-World-s, YOLO-World-m, YOLO-World-l, and YOLO-World-x configurations compared to ground truth, illustrating differences in detection precision and segmentation outcomes.

For ripe wild blueberries (Figure 5), the Swin-B model again demonstrated strong localization accuracy, capturing most individual berries with minimal overlap or misclassification. Swin-T achieved acceptable coverage but with slightly fewer total detections, particularly in dense clusters where multiple berries overlapped. The YOLO-World configurations showed a similar trend, with the smaller variants (YOLO-World-s and YOLO-World-m) detecting fewer instances and the YOLO-World-l increasing recall but occasionally merging nearby fruits under single bounding regions. The comparison highlights that while transformer-based detectors emphasize precise regional boundaries, YOLO-World variants optimized for broader coverage at varying confidence levels.

**Figure 6:**
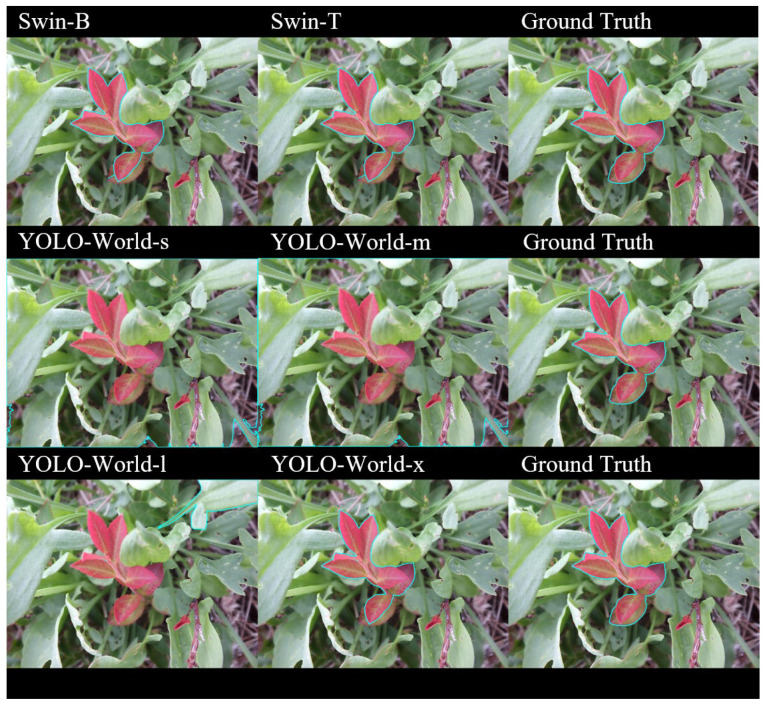
Overlay of segmentation masks (teal) from Swin-B, Swin-T, YOLO-World-s, YOLO-World-m, YOLO-World-l, YOLO-World-x, and ground truth, showing spatial alignment and segmentation accuracy differences.

For leaf segmentation overlays (Figure 6), differences between model–SAM pairings became visually apparent. Both Swin-B and T integrated with SAM2 Large demonstrated superior spatial adherence to leaf boundaries, achieving high fidelity along irregular margins. The YOLO-World models displayed a wider performance range: YOLO-World-s and YOLO-World-m produced sparse masks lacking full contour closure, while YOLO-World-l increased boundary completeness but at the cost of including non-red leaf structures, whereas YOLO-World-x was comparable to the Swin-B and T combinations. These qualitative results align with mIoU findings, confirming that larger detection–segmentation pairs yield more coherent segmentation masks.

**Figure 7:**
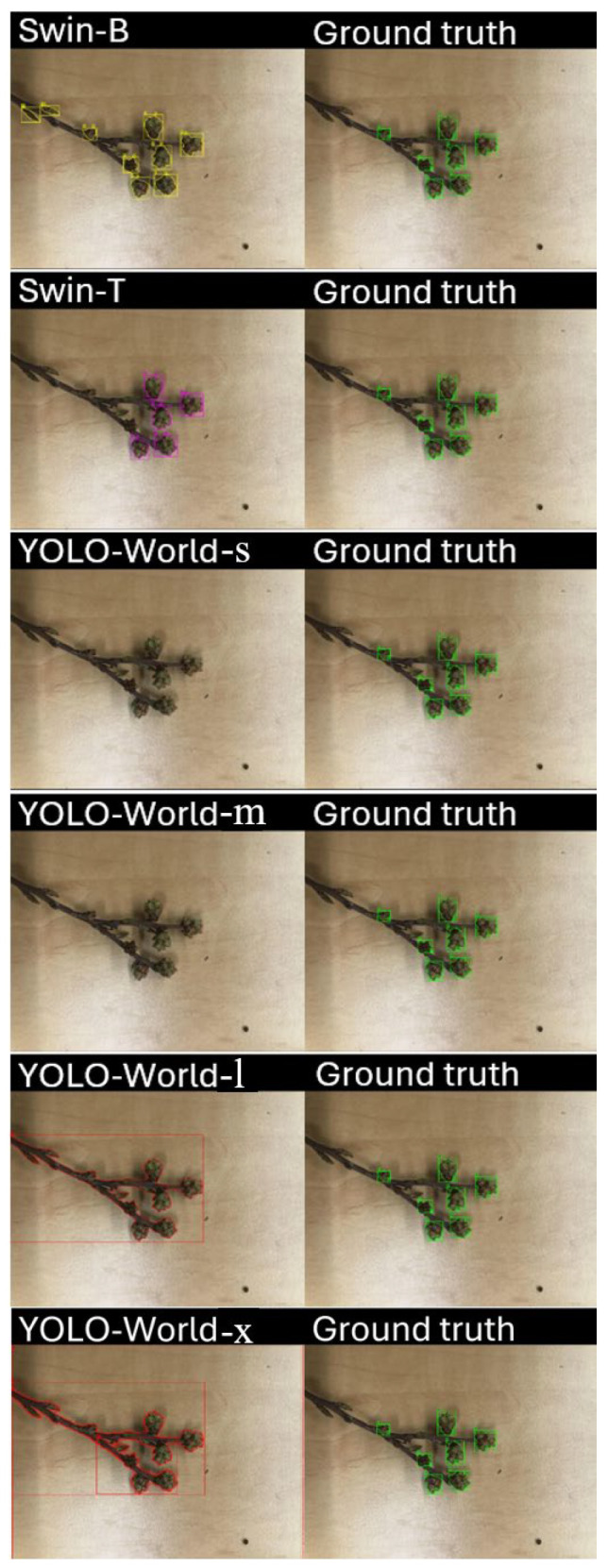
Qualitative comparison of Swin-B (yellow), Swin-T (purple), and YOLO-World models (red) using the same prompt at previously optimized confidence level, demonstrating model-specific differences in detection granularity and region selection.

To further assess prompt-specific sensitivity, all models were evaluated using identical textual prompts and previously optimized confidence thresholds (Figure 7). Under uniform conditions, Swin-B captured nearly all visible buds but also introduced false positives by extending detections to nearby leaf buds. Swin-T produced fewer but more selective detections, avoiding false inclusions yet missing several small targets. YOLO-World-s and YOLO-World-m demonstrated the highest selectivity but lowest completeness, while YOLO-World-l and YOLO-World-x configurations expanded recall at variable precision. This suggests that transformer-based models leverage prompts for broader contextual comprehension, whereas YOLO-World relies more heavily on localized salience, emphasizing the trade-off between generalization and focus.

### 3.2. Failure Analysis

**Figure 8:**
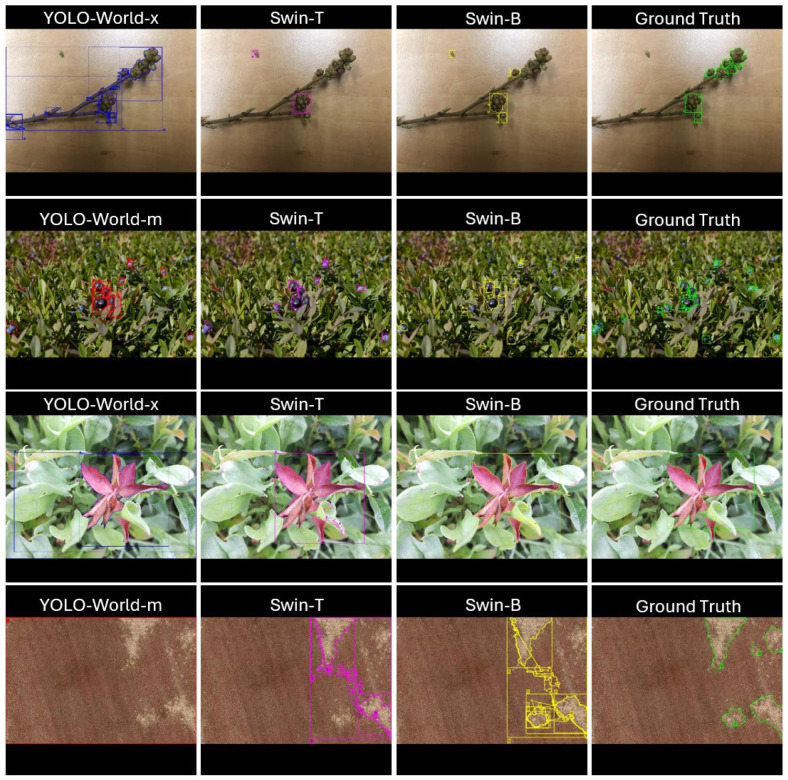
Qualitative comparison of model outputs across four representative tasks: developmental buds, ripe blueberries, red leaf disease, and aerial hair fescue. Columns show predictions from the top performing YOLO-World model (red/blue) (**left**), Swin-T (magenta) (**center**-**left**), Swin-B (yellow) (**center**-**right**), and the ground truth (green) (**right**).

Across the four representative examples, several consistent failure behaviors were observed among the detection–segmentation pipelines relative to the ground truth (Figure 8). The qualitative results highlight clear and recurrent failure tendencies across the different model families. In the developmental bud and ripe blueberry images, YOLO-World variants frequently produced coarse detections that merged objects with surrounding vegetation and overlooked smaller or partially occluded instances. Swin-T demonstrated the opposite behavior, generating clean but conservative segmentations that missed valid targets, while Swin-B improved overall coverage yet still failed to recover peripheral objects and occasionally extended beyond true object boundaries. These patterns reflect consistent model-specific biases, with YOLO-World relying heavily on broad contextual cues and the Swin-based detectors emphasizing localized structure with varying degrees of completeness.

The red leaf disease and aerial hair fescue scenes in Figure 8 further illustrate these differences. YOLO-World again produced oversized detections driven by global chromatic or textural contrast rather than the detailed morphology of the target regions, resulting in substantial over-segmentation. Swin-T and Swin-B localized symptomatic and weedy areas more accurately but continued to struggle with irregular shapes, sometimes introducing small false positives (Swin-T) or fragmenting larger structures into multiple partial detections (Swin-B). These qualitative failures align with the quantitative mIoU trends and the difficulty of achieving reliable fine-scale delineation in agricultural imagery, where dense vegetation, occlusion, and heterogeneous backgrounds pose persistent challenges for both detection and segmentation.

These qualitative analyses provide clear visual evidence of model strengths and limitations, support the quantitative findings, and inform practical selection of model–prompt configurations for various agricultural applications.

## 4. Conclusions

This study integrated zero-shot detection models and SAM2 for automated image annotation, focusing on identifying ripe wild blueberries, F2 developmental buds, hair fescue, and red leaves in wild blueberry fields. It evaluated the performance of Grounding DINO and YOLO-World models with SAM2 to develop an efficient instance segmentation framework. The objectives included applying zero-shot learning for target detection, assessing annotation accuracy, and optimizing model combinations for precision and efficiency.

This study found that Swin-T and Swin-B zero-shot detection models consistently achieved the highest mIoU scores across all SAM2 variants for wild blueberry buds, ripe blueberries, red leaf disease, and hair fescue. SAM2-Large paired with Swin-T achieved the highest mIoU for hair fescue (0.694 ± 0.175) and red leaf disease (0.905 ± 0.114), while Swin-B with SAM2-Large performed best for wild blueberry buds (0.751 ± 0.154). For ripe blueberries, Swin-B paired with SAM2-Small achieved the highest mIoU (0.738 ± 0.189), excelling in complex segmentation. Regarding processing time, SAM2-Tiny, Small, and Base demonstrated the fastest performance with Swin-T (0.30–0.33 s) and Swin-B (0.35–0.38 s). Although SAM2-Large remains within acceptable limits for some real-time applications (0.42–0.48 s with Swin-T and B models), its higher computational demand makes it less practical when in comparison to Small and Tiny. These results highlight the effectiveness of Swin-T and Swin-B models with SAM2 variants for segmenting agricultural targets, particularly when computational efficiency is critical. SAM2-Tiny and Small offer a balance between accuracy and speed, making them ideal for real-time applications, while the choice of SAM2 model can be tailored to operational needs. However, the study’s findings are limited by the specificity of the datasets to particular geographic locations and conditions, which may affect generalizability. Although zero-shot models are designed for broad applicability, performance may vary with different textual descriptions or unseen object categories.

Future research should aim to integrate zero-shot mechanisms into a fully automated annotation pipeline for the wild blueberry cropping system, minimizing manual annotation efforts. Priorities include optimizing Grounding DINO for complex agricultural scenarios, conducting extensive real-world testing across diverse environments to enhance robustness and generalization, and expanding the approach to other agricultural datasets to assess its broader applicability. Exploring additional zero-shot models to improve annotation accuracy and efficiency is also essential. These advancements could significantly enhance agricultural image annotation, improving crop management practices in wild blueberry cultivation and beyond.

## Figures and Tables

**Table 1:** Dataset Summary.

Class	Images	Object Instances	Location	Equipment	Resolution
Developmental buds	72	1266	45.493991°, −62.990759° 45.472361°, −63.631111°	iPhone 8 (Apple Inc., Cupertino, CA, USA)	4032 × 3024
Ripe blueberries	66	2227	45.440839°, −63.542934°	Canon EOS RP (Canon Inc., Tokyo, Japan)	6240 × 4160
Red leaf disease	66	130	45.500750°, −63.107650°	Fujifilm HS30EXR (Fujifilm Corporation, Tokyo, Japan)	4608 × 3456
Hair fescue	20	436	Multiple NS fields	DJI M300 RTK + P1 (SZ DJI Technology Co., Ltd., Shenzhen, China)	8192 × 5460

**Table 2:** SAM2 Variant Comparison adapted from Meta AI GitHub repository [41].

Model	Size (M Parameters)	Speed (FPS, A100)	SA-V Test (J&F)
SAM2 tiny	38.9	91.2	76.5
SAM2 small	46	84.8	76.6
SAM2 base	80.8	64.1	78.2
SAM2 large	224.4	39.5	79.5

**Table 3:** Summary of zero-shot detection mechanism optimization of confidence and prompt inputs across datasets and models.

Framework	Model	Dataset	Prompt	Confidence (%)	mIoU	Precision (%)	Recall (%)
Grounding DINO	Swin-T	Bud	Buds emerging	41.00	56.2	90.91	44.44
	Swin-B		Developing bud	34.00	69.1	78.57	73.33
	Swin-T	Berry	Individual blueberry	36.00	71.6	89.58	48.86
	Swin-B		Blueberry	36.00	68.2	80.33	55.68
	Swin-T	Fescue	Patches of fescue	24.00	80.4	84.21	59.26
	Swin-B		Fescue patches	15.00	75.4	76.00	70.37
	Swin-T	Red leaf	A red leaf disease	40.00	95.3	100.00	71.43
	Swin-B		A cluster of red leaves	30.00	95.5	100.00	85.71
YOLO-World	s	Bud	Bud with leaves	0.12	39.4	42.11	17.78
	m		Bud with leaves	0.70	15.7	16.67	4.44
	l		Bud and sprouting leaves	0.31	31.9	17.58	35.56
	x		Bud with leaves	0.28	44.0	55.26	46.67
	s	Berry	A blue fruit	0.80	55.7	55.77	32.95
	m		Spherical blueberry	0.12	59.4	52.86	42.05
	l		A single, round blueberry	4.00	66.1	56.00	47.73
	x		A blue fruit	1.15	63.8	64.71	50.00
	s	Fescue	Grass spots	0.04	29.3	6.67	3.70
	m		Fescue grass regions	0.01	22.3	0.00	0.00
	l		Fescue grass spots	0.00	29.0	0.00	0.00
	x		Fescue grass regions	0.23	24.7	0.00	0.00
	s	Red leaf	Bright red leaves	3.70	50.0	44.44	57.14
	m		A cluster of red leaves	1.00	34.5	18.18	28.57
	l		A cluster of red leaves	1.40	50.9	2.96	85.71
	x		A cluster of red leaves	1.00	85.5	45.45	71.43

**Table 4:** Mean Intersection over Union (mIoU) performance between SAM2 models and zero-shot detection model variants on developmental wild blueberry bud images (α = 0.05) (L* = 60.04 ± 19.65) (maximum η^2^ = 0.31).

Model	SAM2-Tiny	SAM2-Small	SAM2-Base	SAM2-Large
YOLO-World-s	0.473 (±0.254) abc	0.478 (±0.253) abc	0.447 (±0.279) abc	0.474 (±0.252) abc
YOLO-World-m	0.375 (±0.181) abc	0.360 (±0.096) bc	0.346 (±0.118) c	0.373 (±0.177) b
YOLO-World-l	0.353 (±0.300) c	0.348 (±0.300) c	0.389 (±0.319) abc	0.414 (±0.329) abc
YOLO-World-x	0.610 (±0.178) abc	0.597 (±0.192) abc	0.583 (±0.202) abc	0.586 (±0.205) abc
Swin–T	0.642 (±0.215) abc	0.645 (±0.215) abc	0.641 (±0.212) abc	0.644 (±0.214) abc
Swin–B	0.743 (±0.152) ab	0.748 (±0.155) a	0.744 (±0.152) ab	0.751 (±0.154) a

Metric inside table is mean Intersection over Union with standard deviation in parentheses. Means followed by the same letter are not significantly different according to Tukey’s HSD (*p* ≤ 0.05).

**Table 5:** Mean Intersection over Union (mIoU) performance between SAM2 models and zero-shot detection model variants on ripe wild blueberries images (α = 0.05) (L* = 47.53 ± 19.19) (maximum η^2^ = 0.03).

Model	SAM2-Tiny	SAM2-Small	SAM2-Base	SAM2-Large
YOLO-World-s	0.631 (±0.164)	0.627 (±0.168)	0.631 (±0.154)	0.660 (±0.182)
YOLO-World-m	0.720 (±0.171)	0.706 (±0.172)	0.705 (±0.166)	0.705 (±0.174)
YOLO-World-l	0.704 (±0.204)	0.704 (±0.207)	0.702 (±0.204)	0.705 (±0.203)
YOLO-World-x	0.687 (±0.181)	0.679 (±0.173)	0.675 (±0.178)	0.685 (±0.177)
Swin–T	0.726 (±0.191)	0.728 (±0.188)	0.720 (±0.189)	0.730 (±0.189)
Swin–B	0.731 (±0.200)	0.738 (±0.189)	0.723 (±0.202)	0.738 (±0.194)

Metric inside table is mean Intersection over Union with standard deviation in parentheses.

**Table 6:** Mean Intersection over Union (mIoU) performance between SAM2 models and zero-shot detection model variants on red leaf disease images (α = 0.05) (L* = 60.26 ± 19.55) (maximum η^2^ = 0.46).

Model	SAM2-Tiny	SAM2-Small	SAM2-Base	SAM2-Large
YOLO-World-s	0.466 (±0.419) abcd	0.426 (±0.432) abcd	0.484 (±0.428) abcd	0.425 (±0.440) abcd
YOLO-World-m	0.245 (±0.296) d	0.280 (±0.367) d	0.331 (±0.351) cd	0.351 (±0.367) bcd
YOLO-World-l	0.290 (±0.392) d	0.284 (±0.383) d	0.223 (±0.342) d	0.286 (±0.407) d
YOLO-World-x	0.835 (±0.176) ab	0.885 (±0.097) a	0.812 (±0.245) abc	0.854 (±0.224) a
Swin-T	0.884 (±0.110) a	0.878 (±0.112) a	0.814 (±0.275) abc	0.905 (±0.114) a
Swin-B	0.866 (±0.135) a	0.867 (±0.130) a	0.843 (±0.212) ab	0.887 (±0.131) a

Metric inside table is mean Intersection over Union with standard deviation in parentheses. Means followed by the same letter are not significantly different according to Tukey’s HSD (*p* ≤ 0.05).

**Table 7:** Mean Intersection over Union (mIoU) performance between SAM2 models and zero-shot detection model variants on aerial fescue grass images (α = 0.05) (L* = 44.25 ± 7.36) (maximum η^2^ = 0.77).

Model	SAM2-Tiny	SAM2-Small	SAM2-Base	SAM2-Large
YOLO-World-s	0.115 (±0.077) b	0.115 (±0.077) b	0.113 (±0.080) b	0.112 (±0.080) b
YOLO-World-m	0.127 (±0.083) b	0.126 (±0.082) b	0.113 (±0.080) b	0.112 (±0.080) b
YOLO-World-l	0.115 (±0.077) b	0.115 (±0.077) b	0.115 (±0.077) b	0.115 (±0.077) b
YOLO-World-x	0.115 (±0.077) b	0.115 (±0.077) b	0.112 (±0.080) b	0.113 (±0.081) b
Swin-T	0.651 (±0.217) a	0.660 (±0.226) a	0.681 (±0.231) a	0.694 (±0.175) a
Swin-B	0.606 (±0.227) a	0.626 (±0.240) a	0.647 (±0.231) a	0.647 (±0.223) a

Metric inside table are mean Intersection over Union with standard deviation in parentheses. Means followed by the same letter are not significantly different according to Tukey’s HSD (*p* ≤ 0.05).

**Table 8:** Processing time performance between SAM2 models and zero-shot detection model variants across all datasets (α = 0.05) (maximum η^2^ = 0.46).

Model	SAM2-Tiny (s/Image)	SAM2-Small (s/Image)	SAM2-Base (s/Image)	SAM2-Large (s/Image)
YOLO-World-s	0.59 (±0.10) efg	0.58 (±0.10) efg	0.65 (±0.11) cdefg	0.84 (±0.10) bcdef
YOLO-World-m	0.60 (±0.12) defg	0.60 (±0.10) defg	0.65 (±0.10) cdefg	0.86 (±0.11) abcde
YOLO-World-l	0.97 (±0.54) abcd	1.00 (±0.60) abc	1.03 (±0.57) ab	1.23 (±0.54) a
YOLO-World-x	0.64 (±0.11) cdefg	0.64 (±0.10) cdefg	0.71 (±0.11) bcdefg	0.90 (±0.11) abcde
Swin-T	0.30 (±0.10) g	0.30 (±0.10) g	0.33 (±0.10) g	0.42 (±0.11) fg
Swin-B	0.35 (±0.11) g	0.36 (±0.10) g	0.38 (±0.10) g	0.48 (±0.13) efg

Metric inside table is processing time in seconds/image with standard deviation in parentheses. Means followed by the same letter are not significantly different according to Tukey’s HSD (*p* ≤ 0.05).

## Data Availability

The original contributions presented in this study are included in the article. Further inquiries can be directed to the corresponding author.

## Abbreviations

The following abbreviations are used in this manuscript:

AI	Artificial Intelligence
ANOVA	Analysis of Variance
CLIP	Contrastive Language–Image Pretraining
CPU	Central Processing Unit
GPU	Graphics Processing Unit
HSD	Honest Significant Difference
IoU	Intersection over Union
LLM	Large Language Model
mIoU	Mean Intersection over Union
SAM	Segment Anything Model
SAM2	Segment Anything Model Version 2
ViT	Vision Transformer
YOLO	You Only Look Once

## Appendix A

**Table A1:** F1-score, precision, and recall performance between SAM2 models and zero-shot detection model variants on aerial fescue grass images.

Detection Model	Segmentation Model	F1-Score	Precision	Recall
YOLO-World-s	Tiny	0.198 ± 0.111	0.1144 ± 0.0738	0.9993 ± 0.001
YOLO-World-m	Tiny	0.2165 ± 0.1191	0.1276 ± 0.0799	0.9838 ± 0.049
YOLO-World-l	Tiny	0.198 ± 0.111	0.1144 ± 0.0738	0.9993 ± 0.001
YOLO-World-x	Tiny	0.198 ± 0.111	0.1144 ± 0.0738	0.9993 ± 0.001
YOLO-World-s	Small	0.198 ± 0.111	0.1144 ± 0.0738	0.9993 ± 0.001
YOLO-World-m	Small	0.2151 ± 0.1175	0.1257 ± 0.0777	0.9971 ± 0.007
YOLO-World-l	Small	0.198 ± 0.111	0.1144 ± 0.0738	0.9993 ± 0.001
YOLO-World-x	Small	0.198 ± 0.111	0.1144 ± 0.0738	0.9993 ± 0.001
YOLO-World-s	Base	0.194 ± 0.1156	0.1123 ± 0.076	0.9527 ± 0.1465
YOLO-World-m	Base	0.194 ± 0.1156	0.1123 ± 0.076	0.9527 ± 0.1465
YOLO-World-l	Base	0.1981 ± 0.111	0.1145 ± 0.0738	0.9993 ± 0.001
YOLO-World-x	Base	0.1937 ± 0.1158	0.1122 ± 0.076	0.9507 ± 0.1494
YOLO-World-s	Large	0.1927 ± 0.1164	0.1117 ± 0.0764	0.9424 ± 0.1513
YOLO-World-m	Large	0.1928 ± 0.1163	0.1117 ± 0.0764	0.9431 ± 0.1512
YOLO-World-l	Large	0.1982 ± 0.1103	0.1103 ± 0.0735	0.9961 ± 0.01
YOLO-World-x	Large	0.192 ± 0.1168	0.1113 ± 0.0766	0.9369 ± 0.1538
SwinB	Tiny	0.7295 ± 0.1764	0.6543 ± 0.2311	0.9177 ± 0.1281
SwinB	Small	0.7414 ± 0.1836	0.6637 ± 0.2283	0.9312 ± 0.1317
SwinB	Base	0.7576 ± 0.1802	0.6954 ± 0.2282	0.917 ± 0.1302
SwinB	Large	0.7617 ± 0.1744	0.711 ± 0.2263	0.9037 ± 0.1368
SwinT	Tiny	0.767 ± 0.1579	0.7423 ± 0.1902	0.8345 ± 0.1611
SwinT	Small	0.7716 ± 0.1676	0.7401 ± 0.2022	0.8599 ± 0.1536
SwinT	Base	0.79 ± 0.1666	0.7881 ± 0.1938	0.8449 ± 0.1739
SwinT	Large	0.8173 ± 0.102	0.8206 ± 0.1009	0.8333 ± 0.1528

**Table A2:** F1-score, precision, and recall performance between SAM2 models and zero-shot detection model variants on red leaf disease images.

Detection Model	Segmentation Model	F1-Score	Precision	Recall
YOLO-World-s	Tiny	0.5331 ± 0.3804	0.482 ± 0.4132	0.9759 ± 0.0312
YOLO-World-m	Tiny	0.331 ± 0.2797	0.3277 ± 0.334	0.7925 ± 0.315
YOLO-World-l	Tiny	0.3392 ± 0.3848	0.3218 ± 0.4172	0.6648 ± 0.4397
YOLO-World-x	Tiny	0.8994 ± 0.1127	0.9322 ± 0.1509	0.8944 ± 0.1162
YOLO-World-s	Small	0.486 ± 0.3875	0.4465 ± 0.4326	0.9772 ± 0.0506
YOLO-World-m	Small	0.3481 ± 0.3279	0.4145 ± 0.4082	0.8303 ± 0.3282
YOLO-World-l	Small	0.3346 ± 0.3795	0.3174 ± 0.4114	0.6606 ± 0.4382
YOLO-World-x	Small	0.9364 ± 0.0551	0.9636 ± 0.0488	0.9418 ± 0.082
YOLO-World-s	Base	0.5463 ± 0.3894	0.5227 ± 0.4359	0.9568 ± 0.0909
YOLO-World-m	Base	0.4139 ±0.3363	0.3496 ± 0.3585	0.9744 ± 0.0504
YOLO-World-l	Base	0.2738 ± 0.3446	0.3099 ± 0.4206	0.507 ± 0.459
YOLO-World-x	Base	0.8735 ± 0.1773	0.9175 ± 0.1686	0.8899 ± 0.1989
YOLO-World-s	Large	0.4807 ± 0.3952	0.5252 ± 0.4402	0.8948 ± 0.2709
YOLO-World-m	Large	0.4302 ± 0.351	0.3557 ± 0.3533	0.9927 ± 0.0135
YOLO-World-l	Large	0.325 ± 0.4009	0.3087 ± 0.4183	0.5739 ± 0.4709
YOLO-World-x	Large	0.9011 ± 0.1759	0.9016 ± 0.224	0.9489 ± 0.04
SwinB	Tiny	0.9281 ± 0.0805	0.9824 ± 0.014	0.8885 ± 0.129
SwinB	Small	0.9252 ± 0.0779	0.9724 ± 0.0461	0.8927 ± 0.1248
SwinB	Base	0.9368 ± 0.0769	0.9818 ± 0.0092	0.9045 ± 0.1264
SwinB	Large	0.9391 ± 0.0771	0.9779 ± 0.1253	0.909 ± 0.1177
SwinT	Tiny	0.9401 ± 0.0642	0.9795 ± 0.1037	0.9097 ± 0.1029
SwinT	Small	0.9331 ± 0.0648	0.9682 ± 0.0483	0.9074 ± 0.101
SwinT	Base	0.9377 ± 0.0759	0.9828 ± 0.008	0.9055 ± 0.1256
SwinT	Large	0.953 ± 0.0588	0.9775 ± 0.0265	0.9342 ± 0.0929

**Table A3:** F1-score, precision, and recall performance between SAM2 models and zero-shot detection model variants on developmental wild blueberry bud images.

Detection Model	Segmentation Model	F1-Score	Precision	Recall
YOLO-World-s	Tiny	0.6064 ± 0.2147	0.6532 ± 0.1996	0.6289 ± 0.2835
YOLO-World-m	Tiny	0.5239 ± 0.1629	0.4935 ± 0.2561	0.7784 ± 0.234
YOLO-World-l	Tiny	0.6774 ± 0.1301	0.6021 ± 0.1803	0.8486 ± 0.1659
YOLO-World-x	Tiny	0.7441 ± 0.1271	0.7454 ± 0.1937	0.8187 ± 0.1808
YOLO-World-s	Small	0.6109 ± 0.2184	0.7181 ± 0.1699	0.6103 ± 0.2861
YOLO-World-m	Small	0.5206 ± 0.1043	0.5172 ± 0.2567	0.7896 ± 0.2671
YOLO-World-l	Small	0.6697 ± 0.1395	0.5919 ± 0.1944	0.8559 ± 0.1593
YOLO-World-x	Small	0.7316 ± 0.1401	0.738 ± 0.2119	0.8064 ± 0.1775
YOLO-World-s	Base	0.5711 ± 0.2565	0.6826 ± 0.2464	0.5207 ± 0.2795
YOLO-World-m	Base	0.5025 ± 0.1222	0.4849 ± 0.2432	0.7622 ± 0.2629
YOLO-World-l	Base	0.7255 ± 0.1206	0.7101 ± 0.1863	0.7933 ± 0.1541
YOLO-World-x	Base	0.7181 ± 0.1538	0.8042 ± 0.1875	0.714 ± 0.2103
YOLO-World-s	Large	0.6078 ± 0.214	0.7682 ± 0.2197	0.5255 ± 0.2368
YOLO-World-m	Large	0.5224 ± 0.1642	0.5008 ± 0.2638	0.7717 ± 0.2584
YOLO-World-l	Large	0.7597 ± 0.0954	0.7762 ± 0.1962	0.8208 ± 0.1615
YOLO-World-x	Large	0.7206 ± 0.1546	0.8182 ± 0.1954	0.7122 ± 0.2099
SwinB	Tiny	0.8529 ± 0.1126	0.8948 ± 0.0355	0.8426 ± 0.19
SwinB	Small	0.8542 ± 0.1128	0.8989 ± 0.039	0.8421 ± 0.1903
SwinB	Base	0.8564 ± 0.1156	0.8994 ± 0.0443	0.8443 ± 0.1898
SwinB	Large	0.8612 ± 0.1167	0.9165 ± 0.0441	0.8386 ± 0.1884
SwinT	Tiny	0.7622 ± 0.1654	0.917 ± 0.0583	0.6764 ± 0.2079
SwinT	Small	0.7645 ± 0.165	0.9235 ± 0.0619	0.677 ± 0.2078
SwinT	Base	0.7673 ± 0.1669	0.9253 ± 0.0596	0.6781 ± 0.207
SwinT	Large	0.7698 ± 0.1681	0.9405 ± 0.0624	0.6743 ± 0.206

**Table A4:** F1-score, precision, and recall performance between SAM2 models and zero-shot detection model variants on ripe wild blueberry images.

Detection Model	Segmentation Model	F1-Score	Precision	Recall
YOLO-World-s	Tiny	0.7639 ± 0.1197	0.8295 ± 0.0612	0.7251 ± 0.1815
YOLO-World-m	Tiny	0.8276 ± 0.1113	0.839 ± 0.0805	0.8284 ± 0.155
YOLO-World-l	Tiny	0.8108 ± 0.1429	0.7779 ± 0.1585	0.8497 ± 0.1264
YOLO-World-x	Tiny	0.8029 ± 0.1274	0.7667 ± 0.1276	0.8591 ± 0.1585
YOLO-World-s	Small	0.7599 ± 0.1192	0.82 ± 0.0988	0.7421 ± 0.1957
YOLO-World-m	Small	0.818 ± 0.11	0.8428 ± 0.0895	0.8057 ± 0.1458
YOLO-World-l	Small	0.8106 ± 0.1439	0.7844 ± 0.1606	0.8417 ± 0.1287
YOLO-World-x	Small	0.7984 ± 0.1221	0.768 ± 0.1274	0.8491 ± 0.1543
YOLO-World-s	Base	0.7649 ± 0.1105	0.8444 ± 0.0569	0.7238 ± 0.1823
YOLO-World-m	Base	0.8174 ± 0.108	0.8337 ± 0.0794	0.8118 ± 0.1471
YOLO-World-l	Base	0.8096 ± 0.1424	0.7762 ± 0.1587	0.8494 ± 0.1262
YOLO-World-x	Base	0.7944 ± 0.1258	0.7577 ± 0.1304	0.852 ± 0.1558
YOLO-World-s	Large	0.783 ± 0.1255	0.8631 ± 0.0637	0.7401 ± 0.192
YOLO-World-m	Large	0.8169 ± 0.1128	0.8384 ± 0.0966	0.808 ± 0.1458
YOLO-World-l	Large	0.8121 ± 0.1402	0.7833 ± 0.1571	0.8462 ± 0.1245
YOLO-World-x	Large	0.8027 ± 0.1246	0.7728 ± 0.1259	0.8507 ± 0.1555
SwinB	Tiny	0.8274 ± 0.148	0.7973 ± 0.1915	0.885 ± 0.0996
SwinB	Small	0.8364 ± 0.1387	0.8147 ± 0.1834	0.8811 ± 0.0951
SwinB	Base	0.833 ± 0.1376	0.8041 ± 0.177	0.8836 ± 0.0984
SwinB	Large	0.8405 ± 0.1346	0.8183 ± 0.1767	0.8832 ± 0.0959
SwinT	Tiny	0.8305 ± 0.1242	0.8941 ± 0.0826	0.7865 ± 0.1699
SwinT	Small	0.8341 ± 0.1227	0.9084 ± 0.0778	0.7817 ± 0.1671
SwinT	Base	0.829 ± 0.1254	0.8887 ± 0.0848	0.7874 ± 0.1701
SwinT	Large	0.8358 ± 0.1237	0.9077 ± 0.0816	0.7849 ± 0.1667
